# Resurrecting Epstein–Barr Virus

**DOI:** 10.3390/pathogens11070772

**Published:** 2022-07-06

**Authors:** Roberto Paganelli

**Affiliations:** UniCamillus International Medical University, Via di Sant’Alessandro, 8, 00131 Rome, Italy; roberto.paganelli2@gmail.com

One of the Editor’s choice articles in 2021 published in *Pathogens* was an early assessment of the role of Epstein–Barr virus (EBV) reactivation in the pathogenesis of long-term symptoms associated with COVID-19 [1], a syndrome named long-COVID [1,2,3,4,5]. The syndrome was first recognized among social support groups [6] and only later made its appearance in medical literature, where it has also been named post-acute sequelae of COVID-19 (PASC) or post-acute COVID-19 syndrome (PACS) [5,7]. Its definition is still controversial, due to the heterogeneity of reported symptoms, the different times selected to indicate the persistence of symptoms or emerging ones, and the lack of agreement in terminology [5,7]. The recognition of long-COVID—a term which is preferred by patients [5,6]—was not immediately accepted by the whole medical community [8] because of the wide range of symptoms reported, and this has raised a debate on the underestimation of patients’ perspectives in the assessment of their condition, to the point of questioning medicine’s two cultures [3].

Long-COVID is now largely used by researchers as a very broad umbrella term covering signs, symptoms, and sequelae that continue or develop after acute COVID-19 or SARS-CoV-2 infection for any period of time and are generally multisystemic. The Authors in the study subdivided long-COVID into long- (over 90 days) and short-term (21–90 days) cases, showing these symptoms after recovering from initial SARS-CoV-2 infection: fatigue, insomnia, headaches, myalgia, confusion/brain fog, weakness, rash, pharyngitis, abdominal pain, tinnitus, fever over 101 F, neck lymphadenopathy, or mild-to-moderate hearing loss [1]. The choice of time points is more generous since other studies took 4 weeks after recovery from acute infection or 31 to 300 days from illness onset [9] and at least 12 weeks [10]. The symptoms described are also wide ranging, as in a recent systematic review which found as the most commonly reported symptoms fatigue, dyspnea, sleep disorder, difficulty concentrating, effort intolerance, and myalgia and dyspnea, with substantial between-study heterogeneity for all reported symptom prevalences [10].

As previously noted [8], symptoms occurring after prolonged intensive care unit (ICU) stay are very similar to, but not overlapping with, those presented by long-COVID patients [11,12]. After ICU discharge, they may present persistent cognitive dysfunction, muscle weakness, and distorted memories, as also seen in post-traumatic stress disorder [13,14]. However, it is thought that more than psychiatric and relation problems are present in COVID-19 sequelae, and in fact, long-COVID can be observed in non-hospitalized and even asymptomatic patients, as well as children [15,16,17,18,19]. Therefore, several studies have started to unveil the possible pathogenetic mechanisms of this heterogeneous clinical picture [20]. Epidemiological and clinical studies indicated various co-factors for the development of post-acute symptoms, such as sex, age, obesity, chronic lung disease (including asthma), diabetes, hospitalization, and prolonged viral shedding [2,4,16,17,21,22]. However, mechanistic insights have only recently surfaced, and among them high-level SARS-CoV-2 viremia, EBV reactivation during acute infection, individual gut microbiome profile, and the presence of autoantibodies, in particular those directed against type I interferons (IFNs), have been associated with increased risk of PASC [20,23,24,25,26,27].

Most of the changes seem to persist during follow-up observation, with consistent reports of prolonged viral replication in the gut and viral shedding in feces [28], microbiome alterations [29], presence of autoantibodies and immunological dysfunction [25,30]. The persistence of these factors translates in little improvement of symptoms in patients suffering from long-COVID [15,31]. However, they can be used as markers of risk for developing prolonged manifestations, and some possible treatments are starting to be proposed [32] despite the time needed and difficulty of assessing their effectiveness [33]. Moreover, up to four different subtypes of long-COVID were recognized, each one with slightly different predictors [16,21,24,34]. Many clinical studies have focused on organ damage and pathological sequelae of COVID-19 related to, e.g., neurological, respiratory, or cardiovascular systems. One interesting study [35] has proposed an immunoglobulin signature, based on total IgM and IgG3 levels, which—combined with age, history of asthma, and symptoms during acute infection—can predict the risk of PACS and can do so at any timepoint of blood sampling.

The most important contribution of the Editor’s choice article [1], however, is the early recognition of the pathogenetic role of EBV reactivation in patients with long-COVID. In fact, this had been reported by others in the course of acute infection [36,37,38] and variously interpreted as a possible coinfection or a correlate of disease severity or of immunodepression. The virus was detected either by serology (IgM anti-viral capsid—VCA) or by viral DNA quantitation. In their article, Gold et al. used both VCA-IgM and early antigen-diffuse (EA-D) IgG together with plasma EBV DNA in negative cases, therefore identifying all patients with virus reactivation. Nearly one-third showed a positive test. One possible explanation for these findings is that because of impaired type I IFN activity, partly due to autoantibodies to type I IFNs, patients are less capable of controlling viral quiescence; however, no other herpesviruses, including Cytomegalovirus (CMV), which shares most features of EBV in resurging in immunocompromised hosts, have been detected in these patients, and no study so far has performed both autoantibody and EBV serology tests to study this possibility. Severely immunocompromised patients are more prone to the reactivation of EBV, as also found by Paolucci et al. [36] in COVID-19 cases, and patients with respiratory failure admitted to ICU have been shown to have lower CD8+ lymphocytes counts, more frequent EBV reactivation, and higher mortality [39]. However, no signs of severe immunodeficiency nor ICU admission during acute infection could be held responsible for the findings in the long-COVID cohort studied by Gold et al. [1].

EBV is known to infect over 90% of the world’s population, mostly via asymptomatic transmission during infancy. When infection occurs later, in adolescence or early adulthood, infectious mononucleosis (IM) may develop, with fever, sore throat, lymphadenopathy, and splenomegaly [40]. EBV infects B lymphocytes, establishing also a latent state which persists throughout life and occasionally may give rise to reactivation at times of immunodepression. Although B cell defects including agammaglobulinemia has been reported after IM, this is a feature of a specific primary immunodeficiency, the X-linked lymphoproliferative syndrome, which is caused by SAP deficiency but more frequently results in lymphoproliferative disease, EBV B cell or T cell lymphoma, and hemophagocytic lymphohistiocytosis [40,41]. Many other primary immunodeficiencies predispose to a severe course or cancer development, but they mostly affect T lymphocyte and NK killing functions, with severe CD4 lymhopenia, which results in impaired T and NK cell cytotoxicity, with a massive proliferation of T and NK cells [41,42,43]. EBV is an oncogenic herpesvirus inducing epithelial and smooth muscle tumors. It was discovered in a cell line grown from a biopsy of an endemic childhood cancer in Africa, named Burkitt’s lymphoma [44,45]. Soon it was linked to both IM, other lymphomas and nasopharyngeal and then gastric carcinomas, and its oncogenic potential has no longer been disputed [46].

EBV is the only human virus that induces the proliferative growth of B cells, but in common with other herpesviruses, it establishes in a latent state within infected cells [47,48,49,50,51,52,53,54,55].

Therefore, EBV lives two distinct phases: the lytic and the latent. This latent phase is a sort of truce established between the fighting forces, during which only a small number of viral genes are expressed, and it is a life-long cohabitation, punctuated by rare lytic cycles, mostly asymptomatic. The mechanisms of latency and reactivation have been reviewed in-depth elsewhere [46,47,50,51,52,56,57,58]. It is recognized that reactivation may occur spontaneously [46] or following acute or chronic stress [59], and it is frequently observed in ICU patients [39], where it causes higher mortality. Therefore, it is not surprising that EBV markers of reactivation have been found in hospitalized cases of COVID-19 [36,37,38,60]. This makes sense since a worse outcome in COVID-19 is largely dependent on innate immunity failure, particularly inappropriate type I interferon production [23,36,61,62], and this dovetails with the immunodeficiencies predisposing to severe EBV infection [40,41], which mainly affect the innate immune system.

Another consequence of SARS-CoV2 shared by EBV is the development of autoimmune phenomena after infection. This has been appreciated early in the course of the COVID-19 pandemic [63] after the initial finding of anti-IFN I autoantibodies, which caused a more severe course of infection, delayed viral clearance, and correlated with general immune dysfunction [23,27]. In the case of EBV, reports of its possible involvement in rheumatoid arthritis go back to over 40 years ago [64], and recent reports have renewed interest for its pathogenetic role in several autoimmune diseases [65], particularly rheumatoid arthritis [66] and multiple sclerosis [67]. Despite the many adverse outcomes of EBV infection, and its persistence in a latent state in the majority of adults which poses a continuous threat to human health, very little progress has been made in the development of effective antiviral therapy [46] and in the design of vaccines, both preventive and therapeutic [68]. This is an area which merits careful consideration, since eradication of latent infection would provide a cure for at least 200,000 cancers each year [69] and perhaps protect part of the patients diagnosed with devastating and crippling autoimmune diseases.

Now that some clues on the possible pathogenesis of long-COVID are beginning to surface [20,70], the major culprits seem to be identified, with hyperactivation of coagulation leading to diffuse microvascular thrombosis with organ damage, viral persistence due to neutralization of the host defenses, with inflammatory damages, or prolonged virus-induced immunologic alterations, including autoimmunity and aberrant responses [4,9,17,23,24,25,30,34,36]. To these factors, largely agreed upon, one may add resurrection of EBV from dormancy [1], with the amplification of the most recognized pathogenic mechanisms [39,65].

There is no agreement on the treatment options of patients with long-COVID [33], despite some claims have been made on the use of different drugs. As in many other controversial and new fields, seriously controlled research must guide our quest for a therapy [4,7,71,72].
